# Vesicle Impact Electrochemical Cytometry to Determine Carbon Nanotube-Induced Fusion of Intracellular Vesicles

**DOI:** 10.1021/acs.analchem.1c01462

**Published:** 2021-09-09

**Authors:** Amir Hatamie, Lin Ren, Xinwei Zhang, Andrew G. Ewing

**Affiliations:** Department of Chemistry and Molecular Biology, University of Gothenburg, Kemivägen 10, 41296 Gothenburg, Sweden

## Abstract

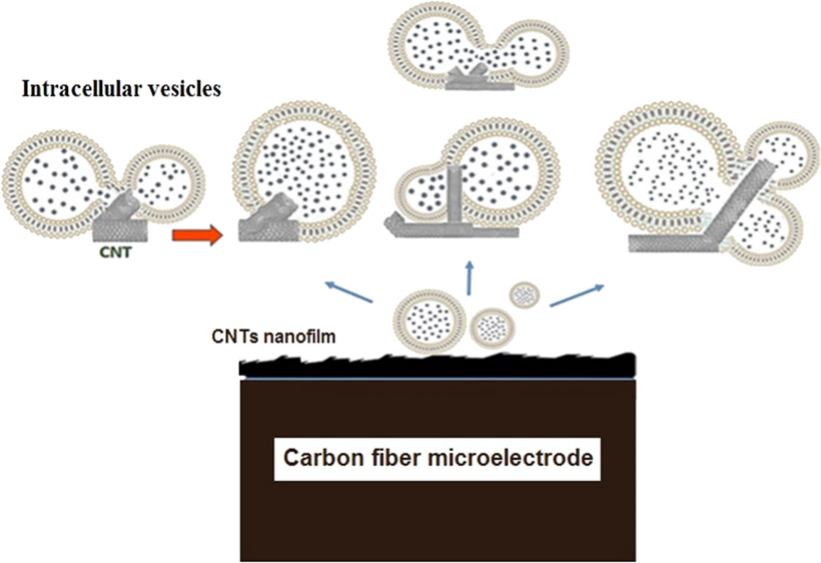

Carbon nanotube (CNT)-modified electrodes are used to obtain new measurements of vesicle content via amperometry. We have investigated the interaction between CNTs and isolated adrenal chromaffin vesicles (as a model) by vesicle impact electrochemical cytometry. Our data show that the presence of CNTs not only significantly increased the vesicular catecholamine number from 2,250,000 ± 112,766 molecules on a bare electrode to 3,880,000 ± 686,573 molecules on CNT/carbon fiber electrodes but also caused an enhancement in the maximum intensity of the current, which implies the existence of strong interactions between vesicle biolayers and CNTs and an altered electroporation process. We suggest that CNTs might perturb and destabilize the membrane structure of intracellular vesicles and cause the aggregation or fusion of vesicles into new vesicles with larger size and higher content. Our findings are consistent with previous computational and experimental results and support the hypothesis that CNTs as a mediator can rearrange the phospholipid bilayer membrane and trigger homotypic fusion of intracellular vesicles.

## Introduction

Membrane fusion is an important process in many biological processes such as cellular trafficking and release of messengers.^1,2^ This process does not occur spontaneously owing to the high energy barrier from biological restrictions such as intermembrane repulsion. However, these energy barriers can be overcome by specific biological interactions with proteins as mediators, which assist the fusion process.^1^ Recently, a few studies have shown that this process can be initiated by nonbiologic agents or mediators such as gold nanoparticles^3,4^ and carbon nanotubes (CNTs).^5^

The introduction of nanomaterials has opened a variety of new applications in many areas such as sensing, separation, electronics, imaging, and cellular drug delivery.^6,7^ Among them, CNTs, owing to their intrinsic high electrical conductivity, specific surface area, and notable mechanical properties, have earned particular attention and have been used in various areas, especially in biomedical and pharmaceutical research.^7−10^ CNTs, acting as a nanovehicle, are a potential choice for drug(s) or biomolecule(s) delivery into cells.^11,12^ Although, the capacity of CNTs for loading (bio)molecules is relatively limited, CNTs can potentially penetrate and cross the cell membrane spontaneously and deliver loaded molecules and drugs into the cell.^13^ Computational^5,14,15^ and experimental microscopy^16−18^ studies have shown that CNTs spontaneously enter into the lipid bilayer of the cell membrane. In spite of the advantages of CNTs as a drug-delivery system, their use raises questions about whether they can interact with the components of cell membranes, lipids, proteins, as well as intracellular organelles, resulting in changes in function or nanotoxicity. CNTs have been shown to interact with surface proteins causing protein displacement;^19^ however, the interactions of CNTs with intracellular organelles such as vesicles have not been as extensively studied.

Intracellular vesicles are nanometer-size structures containing metabolites such as chemical messengers and hormones.^20,21^ CNTs have been detected and imaged in the intracellular space via Raman spectroscopy,^22^ confocal microscopy,^23^ and transmission electron microscopy (TEM).^16,24,25^ However, the effect of CNTs on the chemistry of nanometer vesicles cannot be easily studied by conventional analytical methods owing to the small size and location of both the CNT and the vesicle. Thus, the interaction of CNTs with vesicles has mostly been investigated by using computational methods.

Recent work with molecular dynamics simulations suggests that the presence of CNTs in a vesicle suspension can initiate the spontaneous fusion of lipid vesicles.^5^ Bhaskara et al.^5^ suggest that a single CNT can link two vesicles and perturb their lipid structure, pulling the lipid membranes away and causing a pore to be formed. This leads to the combining of the interior volumes of the two vesicles, and a new vesicle with a larger size is formed. Also, images obtained by confocal microscopy^26^ and cryo-TEM^16^ have shown that CNTs are embedded into the lipid membranes of adjacent vesicles, connecting two separate vesicles, and that CNTs can induce homotypic fusion of individual vesicles. In general, current imaging techniques cannot provide enough information about the chemistry of these phenomena; hence, supplementary analysis of the interactions of CNTs with nanoscale intracellular vesicles requires other chemically sensitive methods.

Amperometry techniques with high temporal resolution provides an approach to measure monoamine molecules (catecholamines,^27−33^ serotonin,^34^ etc.) stored inside or released from single vesicles during exocytosis, and there are reports on the impact of nanomaterials on vesicle trafficking and the exocytosis process. Haynes and co-workers^35,36^ investigated and monitored exocytosis from cells treated with gold and titanium oxide nanoparticles. Their results showed that these nanoparticles can alter the vesicular release of catecholamines during exocytosis from a single cell.

Owing to the nanometer-size structure of vesicles, direct measurement of catecholamine storage has been challenging. A relatively new method, vesicle impact electrochemical cytometry (VIEC),^29,30,32,37,38^ can be used to study the content of intracellular nanoscale vesicles. In VIEC, isolated vesicles are directly adsorbed on a carbon fiber electrode (CFE), where an applied potential (+700 mV vs Ag/AgCl) induces electroporation and vesicle opening.^38^ Oxidative amperometric current transients are then used to quantify the content in each vesicle.

In this paper, we used VIEC to test the hypothesis that CNTs can induce spontaneous homotypic vesicle fusion when CNTs are present on the electrode surface. Furthermore, we examine the analytical ability to use VIEC with CNT-modified electrodes to examine vesicles that undergo homotypic fusion. Quantitatively comparing the amount of catecholamine in isolated vesicles at CNT-modified versus bare electrodes, we found that the presence of CNTs on the electrode surface enhances the amount of catecholamines (*N*) in individual vesicles. This suggests that CNTs act to mediate homotypic fusion or aggregation of adjacent vesicles on the electrode surface.

## Experimental Section

### Chemicals and Solutions

All chemicals and reagents were purchased from Sigma-Aldrich. Aqueous solutions were prepared using 18 MΩ cm^–1^ water from Purelab Classic purification system (ELGA, Sweden). Locke’s buffer (pH 7.4) [56 mM glucose, 56 mM KCl, 1540 mM NaCl, 1% (v/v) penicillin, 36 mM NaHCO_3_, 50 mM HEPES] was made as the stock solution and was diluted 10 times with deionized water the day before the experiment. Homogenizing buffer (pH 7.4) was 10 mM KCl, 230 mM sucrose, 10 mM HEPES, 1 mM EDTA, 0.001 oligomycin, DNase I (10 μg/mL) (Roche), complete enzyme inhibitor (Roche, Sweden), and 1 mM MgSO_4_. The osmolality (osmolality ∼ 317 mOsm/kg) of the homogenizing buffer should be close to the intravesicular lumen to prevent vesicle rupture. CNTs (purity 95%), *D*: 6–13 nm and *L*: 6–13 μm, were obtained from Sigma-Aldrich, Germany. To purify CNTs and increase their active surface area, 100 mg of CNTs were suspended in 20 mL of concentrated HNO_3_ solution for 24 h at 30 °C.^39^ After acid treatment, the suspension was filtered and rinsed with deionized water three times. The filtered CNTs were dried at 100 °C for 24 h.

### Vesicle Isolation

Isolation of vesicles (Figure S1) was carried out based on a reported protocol.^40,41^ Fresh bovine adrenal glands were obtained from a local slaughterhouse (Dalsjöfors Meat AB, Borås—Sweden). To remove the blood cells, the gland samples were washed with Locke’s buffer. The medulla was isolated from each gland and transferred to the homogenizing buffer and homogenized with a homogenizer (Wheaton, U.S.A.) at 4 °C. To purify vesicles, a series of centrifugation steps were performed at 4 °C (Figure S1). First, each sample was centrifuged at 1000*g* for 10 min to remove the tissues, and then the supernatant solution was centrifuged at 10,000*g* for 20 min to pellet the vesicles. The pellet includes isolated vesicles (average vesicle radius ∼ 170 nm)^37^ and was suspended in the homogenizing buffer and then used for electrochemical measurements on the same day.

### Microelectrode Modification and Characterization

The CFEs (33 μm diameter) were fabricated as described previously.^42−44^ (The fabrication method is described in detail in the Supporting Information.) Many well-established techniques have been developed to modify microelectrodes with CNTs.^45−48^ Here, we employed a simple electrophoretic method^49^ as a faster and more direct means to deposit CNTs on 33 μm carbon fiber disk electrodes.

### Electrophoretic Deposition Method

Prior to each deposition, CFEs were electrochemically pretreated and activated in H_2_SO_4_. Each CFE was dipped into a H_2_SO_4_ solution (0.05 M), and cyclic voltammetry (potential window: 0.0 to +1.0 V vs Ag/AgCl, scan rate: 100 mV/s, number of cycles: 10) was applied until a steady voltammogram was achieved. To deposit CNT films, 1.0 mg of cleaned CNTs was dispersed in 20 mL of pure anhydrous dimethylformamide and sonicated for 8 h. To reduce the possibility of CNT aggregation, CNTs were sonicated for at least 1 h prior to each deposition.^49^ A platinum wire (0.5 mm), used as a counter electrode, was fixed parallel at a distance of a few millimeters from the activated CFE in CNT suspension. Finally, a DC electric voltage (0.9 V, 100 s) was applied (CH Instruments, Inc. Austin, USA). Prior to electrochemical tests, to remove the CNTs with weak attachment, the modified electrodes were washed gently with water, and afterward, the CFEs modified with CNTs (CNTs/CFE) were transferred to an oven horizontally (30 min, 100 °C) to evaporate the remaining solvent and increase the stability of the CNT film. SEM analysis showed that the CFE surface was uniformly covered with a thin layer of CNTs. Finally, prior to each VIEC analysis, each CNT/CFE was tested by cyclic voltammetry in a solution of dopamine (100 μM in PBS pH 7.4, −0.2 to 0.8 V vs Ag/AgCl, scan rate: 100 mV/s), and only modified CFEs showing acceptable steady-state currents by CV were chosen for further measurement (Figure S2).

### Data Analysis

Current spikes were recorded by using a Digidata 1440A (Molecular Devices; San Jose, CA) system, filtered at 2 kHz using a 4-pole Bessel filter. Data were converted in Matlab software (The MathWorks, Inc.) and analyzed with IgorPro software (Wavemetrics, Lake Oswego, OR) (details in the Supporting Information). For statistical analysis, all recorded spikes were analyzed, and the median was used as a statistical analysis tool as it is less sensitive to extremes in a non-Gaussian distribution. Medians from all samples were combined, and groups were statistically analyzed with the Mann–Whitney rank sum test (unpaired and two-tailed) using Prism 7 (GraphPad, La Jolla, CA).

## Results and Discussion

### CNT-Coated Electrode Characterization

**Figure 1:**
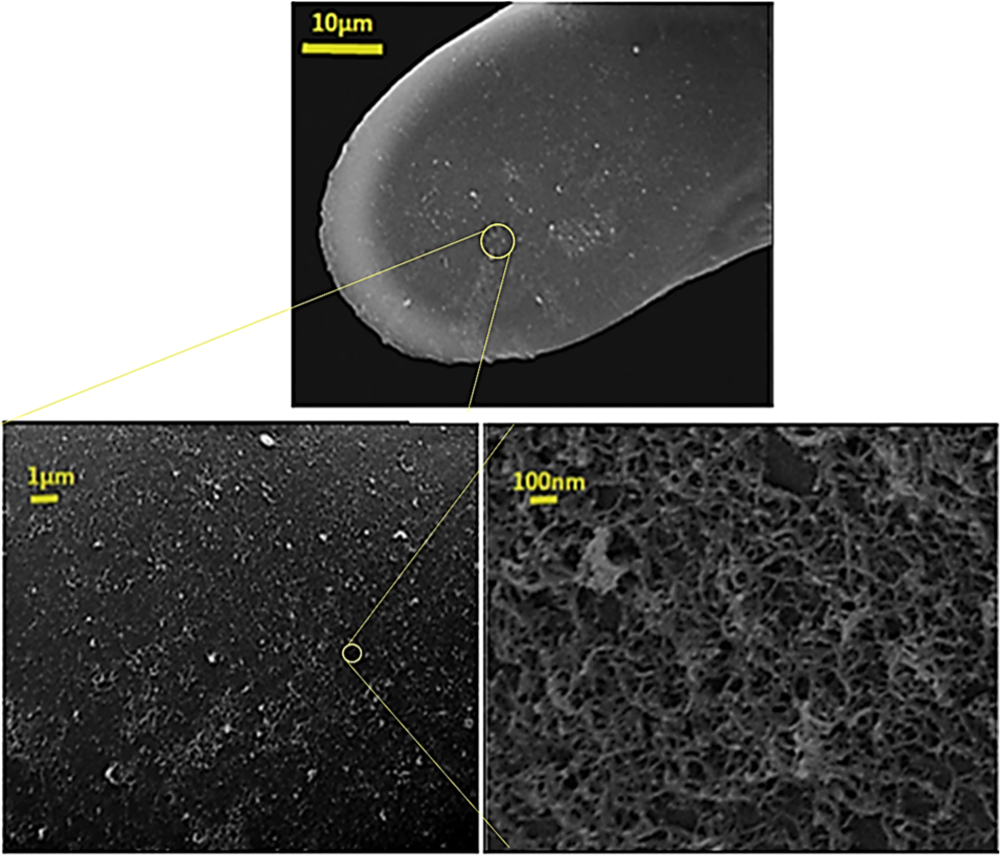
SEM images show the electrodeposited CNTs film on the CFE (33 μm) surface using the electrophoretic method at different magnifications [constant potential: +0.9 V, for 100 s and Pt wire (as a counter electrode)].

Figure 1 shows the electrode surface after modification with CNTs at different magnifications. SEM images (procedural details in the Supporting Information) show that the CFE surface was covered by a uniform nanofilm of CNTs. Figure S2 shows the cyclic voltammograms of bare CFEs and CNT/CFEs following different deposition times in dopamine solution (100 μM in PBS 7.4). The electrode responses and steady-state electrochemical behavior are proportional to the deposition time. Based on the electrophoretic procedure, increasing the deposition time increases the amount of CNTs and the CNT layer thickness as well as the charging current.^50,51^ As can be seen in Figure S2, the anodic peak increases, indicating that the modification increases the electroactive surface area and improves the sensitivity of dopamine oxidation at the CNT/CFE. At the same time, a longer deposition time increases the large background during the VIEC analysis. To minimize the background current, the effect of electrodeposition time was investigated and optimized; after evaluating the modified electrodes, a 100 s deposition time was chosen for the best signal-to-noise ratio and applied for further experiments.

### Vesicle Fusion in the Presence of CNTs

The VIEC technique^38,42,43^ was employed to study the interaction of vesicles with CNTs on CNT/CFEs. VIEC is a sensitive and suitable technique as it can measure the whole vesicle content with high accuracy.^29,31,43^ We have previously established by comparison of simulation to the experiment that we measure the entire contents of each vesicle.^43^ This is expected as the diffusional theory for small electrodes has established the electrode as a diffusional sink for scanning electrochemical microscopy.^52−55^

**Figure 2:**
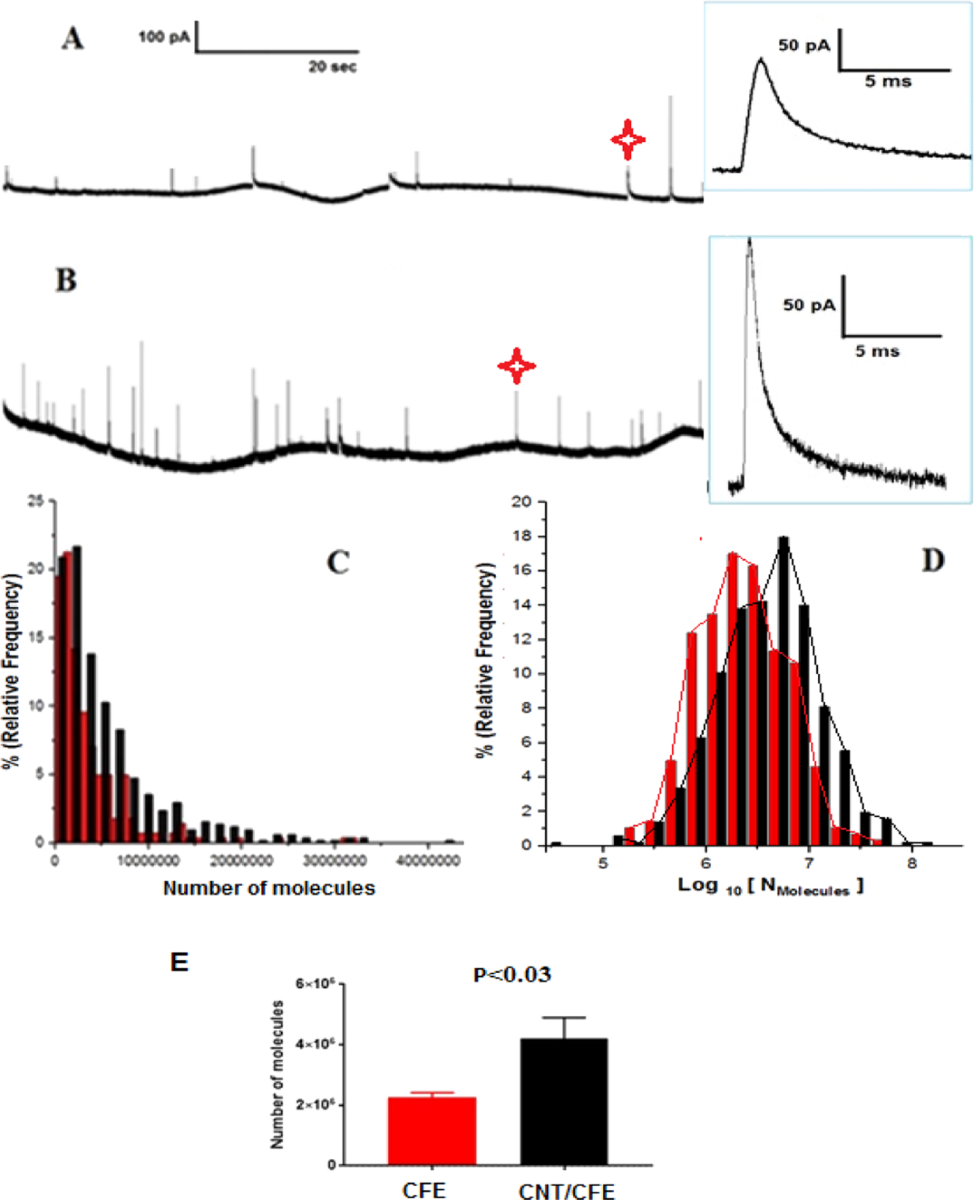
Typical amperometric traces for isolated chromaffin vesicles obtained with VIEC at different electrodes with (A) a CFE and (B) a CFE coated with CNTs (constant potential amperometry: +700 mV vs Ag/AgCl, *t* = 5 min). The enlarged images of a typical amperometric spike marked in each amperometric trace. (C) Normalized frequency histograms describing the distributions of the molecules quantified by VIEC measurements at CFEs (red) and CNT/CFEs (black). (D) Normalized frequency histograms of log[molecules] obtained from VIEC measurements, CFE (red) and CNTs/CFE (black). (E) Comparison of molecules quantified from isolated vesicles with VIEC measurements by using CFEs coated with CNTs and bare CFE (the data are presented as mean of medians). The pairs of data sets were compared using a Wilcoxon–Mann–Whitney test, and the result is indicated next to the variation. Error is the standard error of the mean.

To carry out VIEC experiments (Figure S3), the microelectrodes with or without CNTs were directly dipped into a freshly prepared vesicle suspension while being held at a potential of 700 mV (vs Ag/AgCl electrode). The suspended nanoscale vesicles randomly deposit on the electrode surface, rupture, and release the vesicular catecholamines in a few milliseconds. Current spikes are observed from the rupture of single vesicles, and the catecholamine molecules present in each vesicle are oxidized on the electrode surface and can thus be quantified.^47^ Typical amperometric traces for bare CFEs and CNT/CFEs are shown in Figure 2A,B. The number of molecules (*N*) was obtained by integrating the area under the peak (Figure S4) and by the use of Faraday’s law (*N* = *Q*/*n*F), where *Q* is the area under the peak, *F* is Faraday’s constant, and n is the number of electrons produced during the oxidation reaction (*n* = 2 for catecholamines).^29,37,42−44^

**Figure 3:**
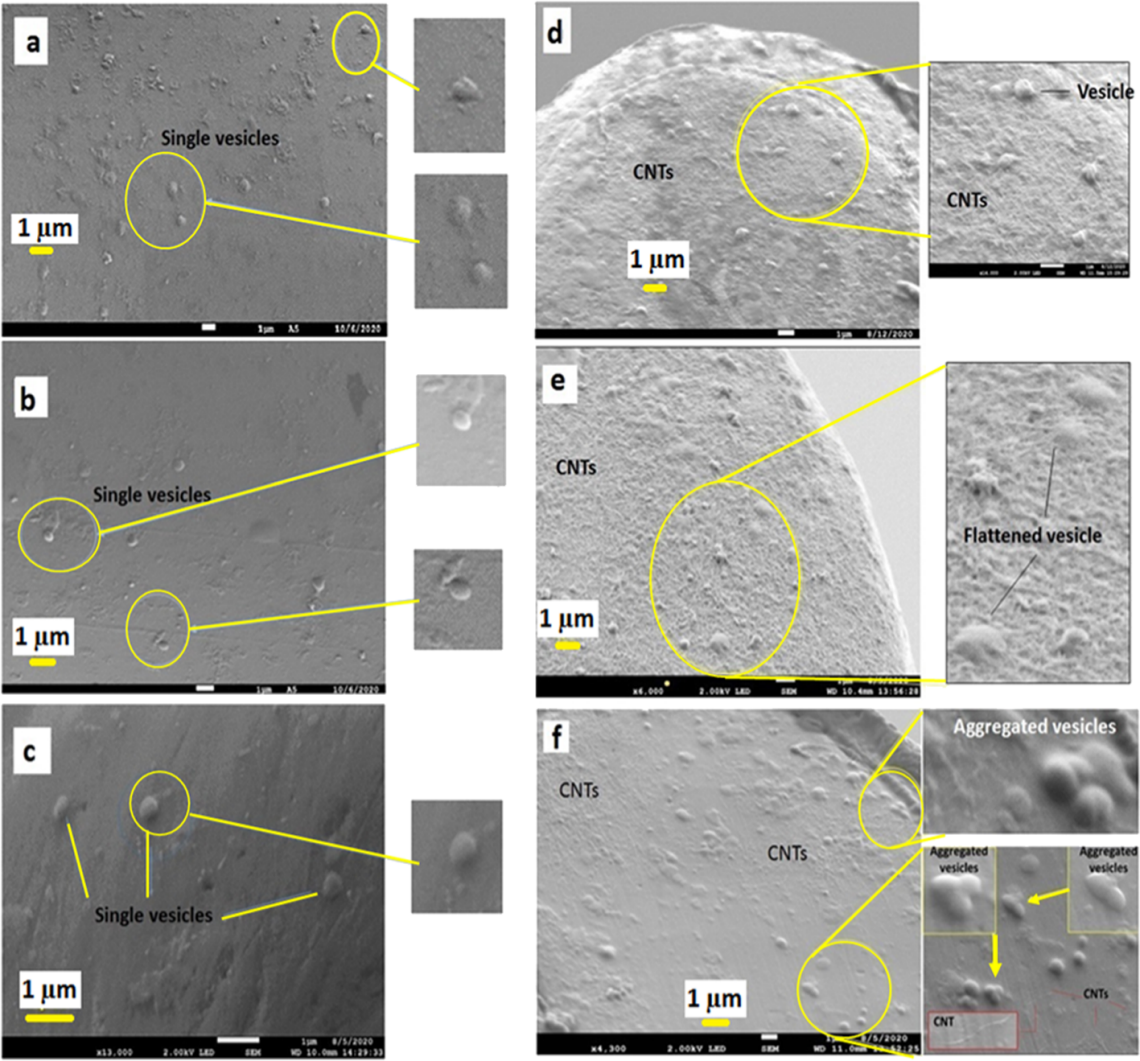
SEM images of adsorbed chromaffin vesicles on the CFE surface (a–c) and CNTs/CFE surface (d–f).

The average spike area increased for electrodes coated with CNTs compared to the bare electrodes, resulting in a higher number of molecules released in the distribution of event size for VIEC at CNT-modified electrodes (Figure 2C,D). When the total vesicular catecholamine content between the modified and unmodified electrodes is compared (Figure 2E), the number of molecules for each event for CNT/CFEs (3,880,000 ± 686,573 molecules, mean of medians ± standard error of the mean, *n* = 9) was nearly double that for CFEs (2,250,000 ± 112,766 molecules, mean of medians ± standard error of the mean, *n* = 9). As the only difference in the experiments is the presence of the CNTs, it appears that the increase in the number of catecholamine molecules results from interaction between CNTs and adsorbed vesicles. Moreover, we took the SEM images of adsorbed vesicles on the electrode surface. As shown in Figure 3, the vesicles adsorbed on the CFE individually, that is, without aggregation. However, on the CNTs/CFE, we found that vesicles in the presence of CNTs are aggregated or fused.

Previous experimental and computational studies have shown that CNTs with a tube structure and hydrophobic properties can adsorb onto, pierce, and pass or remain in the membranes of cells and artificial cells, resulting in deformation and perturbation of the phospholipid bilayer.^13−16^ For vesicles with similar membranes but with smaller size, in addition to adhesion and penetration, aggregation or fusion of membrane of vesicles is also possible.^5,26^

Membrane fusion generally requires proteins in normal biological processes. However, recent reports indicate that some nanostructures like gold NPs^3,4^ and CNTs have the potential to initiate and participate in membrane fusion.^5^ Recently, Geng et al. studied^16^ the interaction between CNTs and lipid vesicles (diameter ∼ 200 nm) by Cryo-TEM. The images suggest that CNTs can rupture and readily cross the membrane, causing vesicle membrane deformation or a change in the vesicle shape, and they can also form a bridge between vesicles. Pérez-Luna et al.^26^ also observed that CNTs could cause deformation and, in some cases, aggregation of unilamellar vesicles; confocal images clearly show two or three vesicles fused or combined with each other. It is possible that under this condition their content can be combined. Based on these observations, Bhaskara et al.^5^ carried out comprehensive molecular dynamics simulations and found that a single hydrophobic CNT between two vesicles can interact with membrane components and lead to rearrangement. Ultimately, this interaction can cause fusion of two distinct vesicles forming a new single vesicle with larger size and combined contents. With regard to the dynamics of the process, further molecular dynamics simulations showed that the fusion process can occur in microseconds.^5^

Based on reported microscopic images and simulation studies showing that vesicles promote fusion in the presence of CNTs^5^ and our VIEC (Figure 2E) and SEM image (Figure 3) results, we suggest that the increased number of molecules in the vesicles observed with VIEC results from the formation of new vesicles on the CNT/CFE surface. This appears to be due to CNT-mediated fusion of attached vesicles on the CNTs located on the electrode surface.

### CNTs Increase VIEC Current Intensity via Variations in Membrane Properties

The maximum peak current during VIEC is related to how the vesicle opens.^39^ Vesicles adsorb to the electrode and are electroporated, opening a small pore in the membrane. As catecholamine diffuses out of the vesicle, the amplitude of the current transient is proportional to the size of the pore. Pore size is in turn related to the membrane properties.

**Figure 4:**
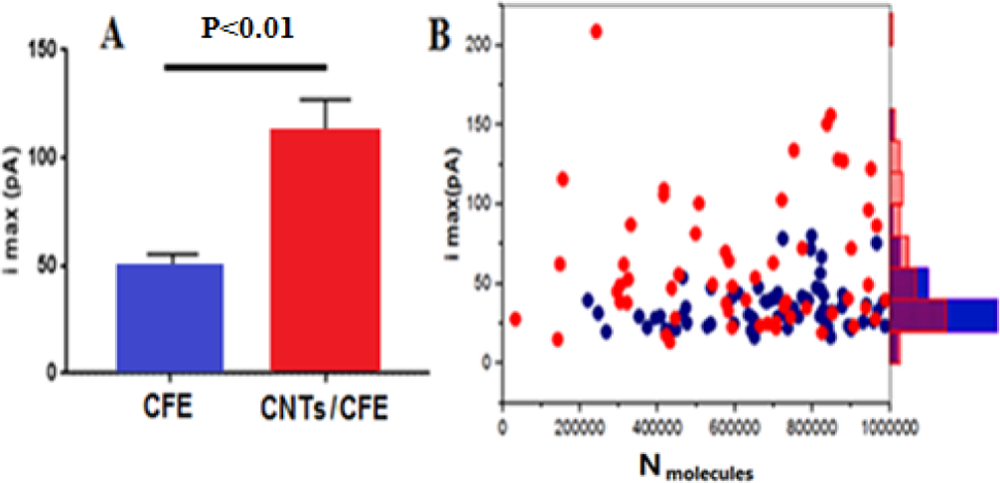
(A) Comparisons of peak current from VIEC measurements by CFE and CNT-coated CFE microelectrodes; error is the standard error of the mean. The pairs of data sets were compared using Wilcoxon–Mann–Whitney test, and the result is indicated next to the variation. (B) Comparisons of peak currents of small vesicles (contain zero-one million catecholamines) from VIEC measurements is shown in a dot plot (red: CNTs/CFE, blue: CFE) and the normalized frequency histograms of peak currents in matching color on the right.

We investigated the amperometric spike amplitudes for both bare and CNT electrodes and observed a significant increase in the maximum current intensity (*I*_max_) (peak parameters are defined in Figure S3). As can be seen in Figure 4A, the *I*_max_ for VIEC at CNT/CFEs (108 ± 16 pA, mean of medians ± standard error of the mean, *n* = 9) was essentially double that observed at CFEs (48 ± 4.5 pA, mean of medians ± standard error of the mean, *n* = 9). Previous studies^37,42^ suggested that *I*_max_ mainly depends on the pore size. Membrane structure and composition, electrode potential, electrode material, and electrode surface topography at the contact point might also be factors that alter the electroporation process.^44^ We evaluated whether the *I*_max_ enhancement for CNT/CFEs is dependent on the vesicle size.

We compared *I*_max_ for small vesicles containing 0.0–1.0 million catecholamine molecules at both modified and unmodified microelectrodes, and data are shown in Figure 4B. Molecular dynamics simulations^5^ suggest that fusion of vesicles in the presence of CNTs is significantly less likely for smaller versus larger vesicles. This is consistent with the range of catecholamine molecule contents observed (Figure 2C). Interestingly, the VIEC results are consistent with the simulation result^5^ as considerably significantly more vesicles with the same or close number of catecholamines observed with CNT/CFEs have higher *I*_max_ (with the same area or charge). So, we hypothesize that the formation of increased vesicle size owing to CNT-induced fusion and the interaction of vesicle membranes with the CNTs also alters and facilitates the electroporation process to enhance pore opening and *I*_max_ intensity. In addition to *N*_molecules_ and *I*_max_, the dynamics of VIEC signals (Figure S4) have been affected. As can be seen in Figure S5, *t*_rise_, *t*_1/2_, and *t*_fall_ decrease in the presence of CNTs.

### Hypotheses to Explain VIEC Charge and Current during VIEC

**Figure 5:**
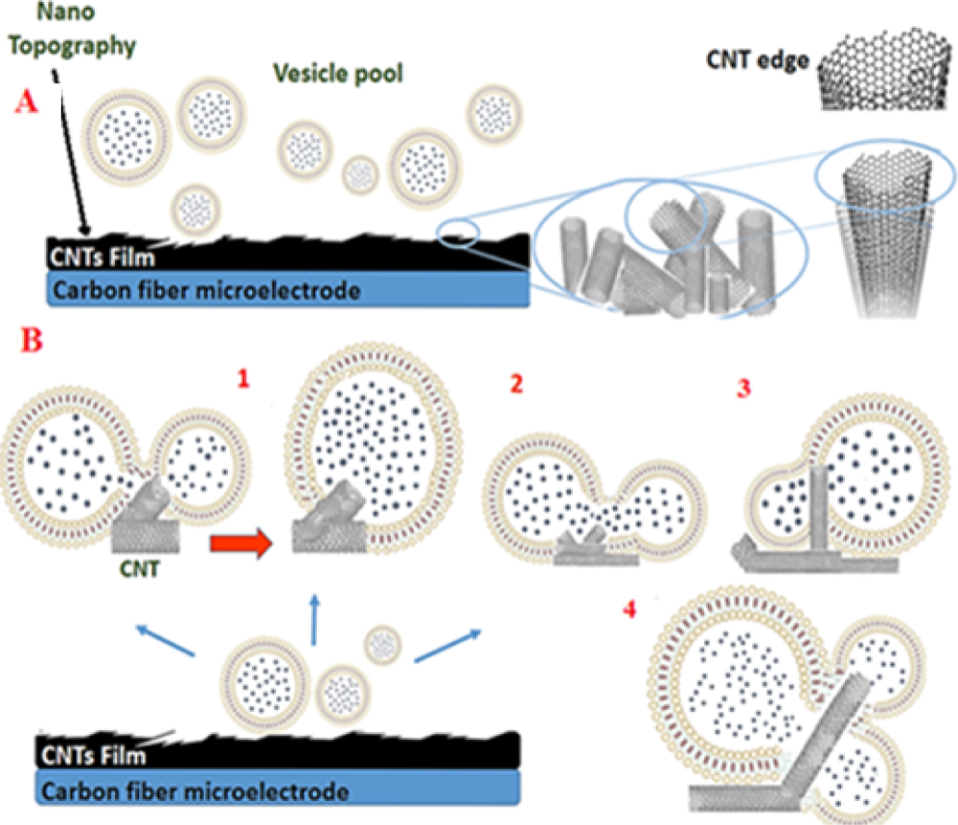
(A) Schematic of topological features possible on a CFE surface in the presence of CNTs and vesicles. (B) Four possible models for fusion or aggregation of vesicles. Based on the literature,^5,16,26,57^ we suggest in (B1–4) that CNTs might serve as intermediates to cause complete or incomplete fusion between two or more vesicles.

Based on our findings and relying heavily on reported studies,^5,16,26^ we hypothesize three possible mechanisms to explain how the vesicles might fuse or aggregate in the presence of CNTs (Figure 5) and how these interactions can lead to fusion and formation of a new single vesicle with different shapes and sizes and higher catecholamine content as detected by VIEC. First, a single CNT might serve as an intermediate to induce complete fusion and aggregation of adjacent adsorbed vesicles (two vesicles or more) and produce a new vesicle with a larger size (Figure 5B-1). Second, incomplete or partial fusion might occur where the CNT induces incomplete fusion by allowing partial mixing (Figure 5B-2,3). Finally, multiple fusion events might occur where three or more vesicles aggregate and mix their contents (Figure 5B-4). Furthermore, we analyzed the electrochemical signals to find the percentages of single spikes and multiple spikes (double and triple) resulting from incomplete or multiple fusions. We find that a majority of events include individual single spikes (Figure 2A,B) with high intensity (*I*_max_), and double or triple spikes are rarely observed indicating that full fusion is predominant. Also, this shows that a scenario where two or more vesicles are detected by one CNT in parallel is not highly likely.

Based on reported studies^5,26,56,57^ about the interaction of CNTs with membrane components and our electrochemical and imaging analysis, we suggest possible interactions that can lead to CNTs altering the vesicle membrane properties, stability, and finally the electroporation process, which are summarized in Figure S6. Recently, the effect of the CNT films as a surface substrate with a nonporous morphology on cell membranes was studied by Alexander-Katz et al.^56^ Their results suggested that a CNT film with nanoroughness can cause changes in the nanoscale membrane curvature, bending, and thickness and increase lipid spacing in the membrane, especially at the CNT edges. Consequently, the stability of the cell membrane is decreased remarkably when deposited on the CNT/CFEs. As the cell membrane structure is highly similar to the vesicle membrane, this work might be useful to model the interaction between CNTs on the electrode surface and vesicle membranes. We assume the stability of the vesicle membrane is decreased owing to the nano roughness of the CNTs enhancing electroporation.

CNTs also have a highly hydrophobic surface leading to strong adsorption of the phospholipid bilayer on them.^56,57^ We suggest that the strong interaction between CNTs and the membranes of the adsorbed vesicles might change them from a spherical shape to an ellipsoidal shape. This leads to stretching and reduction of the thickness of the membrane, reducing its stability. To support this idea, recently, stretched cell membranes and varied cell shapes were identified when attached on deposited CNT nanofilms.^58−60^ Moreover, Sheng et al.^57^ carried out a comprehensive theoretical study which showed that adhesion of a single vesicle onto a hydrophobic surface (similar to a CNT film on an electrode surface) leads to a significant deformation of the vesicle shape from a spherical shape to an ellipsoidal shape (Figure S6). Previous reports^19,60^ have suggested that the molecular components of the cell membrane such as lipids and proteins at the contact point can be dislocated from the cell surface by CNTs, and this might affect the size of the pore formed in the membrane during VIEC.

We believe that another important factor affecting the electroporation process might be surface proteins on the membrane. Our group has speculated the presence of surface proteins on the membrane which can inhibit electroporation in VIEC.^44^ These proteins produce a distance between the membrane and the electrode surface, forming a barrier and decreasing the contact point area. Thus, movement of proteins (Figure S6) prior to electroporation is necessary. Interestingly, based on the previous reports, CNTs can cause these physical movements.^19,56^ So, at CNT/CFEs, the CNTs might adsorb these surface proteins and cause diffusion of the proteins away from the contact area and thus reduce the distance between the lipid bilayer and the electrode.^40^

Previous reports^60^ indicated that the amount of protein movement at a randomly oriented CNT surface (similar CNT/CFE surface) is higher than for bare surfaces. This movement allows the lipid bilayer to move closer to the electrode, thus increasing the electric field across the membrane to reach the electroporation threshold.^44^

Another possibility that can decrease the stability of the cell membrane is the penetration of CNT edges into the vesicle membrane (Figure S6). As reported, the sharp and thin edges of CNTs have high potential that can penetrate into the vesicle membrane easily and deform the cell membrane physically.^59^ We suggest that penetration of the sharp edges of deposited CNTs into the vesicle membrane on the modified electrode might decrease the stability of the membrane and facilitate the electroporation process.

## Conclusions

In this work, the content of nanoscale single vesicles isolated from chromaffin cells was quantified on CNT-modified and unmodified microelectrodes. A significantly increased vesicular catecholamine content at CNT/CFEs suggests strong interactions between CNTs on the surface and the vesicle membrane where the CNTs with a needle-shaped structure and a highly hydrophobic surface serve as mediators and can directly initiate vesicle fusion or aggregation during the adsorption and even perhaps the vesicle opening process. The VIEC results are consistent with previous computational and experimental results and support the hypothesis that CNTs can also serve as mediators and trigger the fusion of the vesicles. Moreover, our data are also consistent with previous reports that claimed CNT films can interact with vesicle membranes and their components’ changing membrane structure and properties. As a result, the membrane stability is decreased, the attachment area of the membrane is increased, and an enhancement of the VIEC peak current is observed. In addition to quantification of vesicle contents, VIEC might be used to analytically study the aggregation or fusion of nanoscale vesicles. A final note, the experiments here show that VIEC provides the means to better understand the applications of CNTs in biological systems such as nanocarriers and nano-based drug-delivery systems.

## Supporting Information Available

Additional experimental details and figures, microelectrode fabrication and SEM analysis procedure, schematic illustrating the procedure for isolating chromaffin vesicles, representative cyclic voltammograms from a bare CFE and a modified CFE, and schematic of the experimental process of VIEC analysis (PDF)
