# Supplementary material for: Vesicle Impact Electrochemical Cytometry to Determine Carbon Nanotube-Induced Fusion of Intracellular Vesicles
Source: Anal Chem. 2021 Sep 9;93(39):13161–8. doi: 10.1021/acs.analchem.1c01462 (PMC8495673; doi:10.1021/acs.analchem.1c01462)
Supplement: Supplementary file 1 — ac1c01462_si_001.pdf [file ac1c01462_si_001.pdf]

Supporting Information

Vesicle Impact Electrochemical Cytometry to Determine Carbon Nanotube-Induced Fusion of Intracellular Vesicles

Amir Hatamie,[1] Lin Ren,[1] Xinwei Zhang,[1] Andrew Ewing [1]*
Department of Chemistry and Molecular Biology, University of Gothenburg, Kemivägen 10, 41296 Gothenburg, Sweden.

Table of Contents:

■ Microelectrode Fabrication.
■ SEM analysis.
■ Data Analysis.
■ Figures

Microelectrode Fabrication.

Carbon fiber electrodes (CFEs) were fabricated based on previous reports.[1-2] Briefly, a 33-µm diameter carbon fiber was placed into a borosilicate capillary (1.2 mm o.d., 0.69 mm i.d., Sutter Instrument Co., Novato, CA, U.S.A.) by aspiration. A micropipette puller (Model P-1000, Sutter Instruments Co., Novato, CA, U.S.A.) was used to pull each capillary. The capillary tip was sealed by dipping in a solution of epoxy (Epoxy Technology, Billerica, MA, U.S.A.). All electrodes were then kept at 100 °C for 24 h for curing, subsequently cut at the glass junction and beveled at a 45° angle by using a beveller (EG-400, Narishige Inc., London, U.K.). Prior to the electrochemical measurement, each electrode was tested by cyclic voltammetry in a standard dopamine solution (100 µM) in PBS pH 7.4 (−0.2 to 0.8 V vs Ag/AgCl, at scan rate 100 mV/s) and only microelectrodes showing acceptable steady-state currents at CV were accepted and used.

SEM analysis.

SEM images of modified electrodes were obtained using a SEM (JEOL model 7000F, resolution of 1.2 nm, accelerating voltage of 2-5 kV, working distance of 10 mm). At first the electrode was coated with a palladium nano film, then the coated CNT/CFE was put inside the SEM sample holder, and then the SEM images were obtained.

For vesicle imaging, both electrodes were placed in a concentrated chromaffin vesicle stock solution for 15 min at 4 °C to allow adsorption of vesicles on the electrodes surface. After the electrodes were loaded with vesicles, the electrode was gently rinsed with PBS, dipped in formaldehyde solution (4%) for 15-20 min to fix the vesicles, and dried at room temperature.[1] SEM images (Figure 3) were taken as described above.

Data and peak analysis.

Amperometric signals were converted in Matlab software (The MathWorks, Inc.) and analyzed with IgorPro software (Wavemetrics, Lake Oswego, OR). A binomial filter and the detection limit was set to 1 kHz and five times the standard deviation of the noise measured as the threshold for peak detection. Also, all amperometric traces were checked manually and after peak detection, the false positives were manually removed. Each spike as a representative of one vesicle was analyzed using Faraday's law ($N=Q/nF$), where Q is the area under the peak (Figure S4), N, number of catecholamine molecules, obtained by integrating the area under the peak, F is Faraday's constant and n is the number of electrons produced during the oxidation reaction (n=2 for catecholamines). Also each spike was analyzed by the following parameters (Figure S4). Imax (pA), the peak current intensity, t_{rise} (ms), rise time, defined as the time that takes for the current to increase from 25% to 75% of I_{max}, $t_{1/2}$ (ms), the half peak width at half-maximum Imax, t_{fall} (ms), fall time, defined as the time that takes for the current to drop from 75% to 25% of I_{max}, (Figure 4S). [3-4]

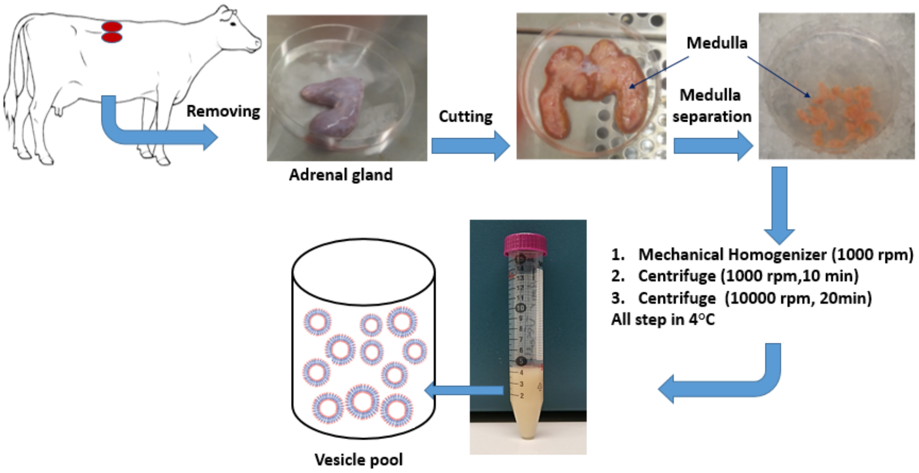

Figure S1. Schematic illustrating the procedure for isolating chromaffin vesicles from adrenal glands.

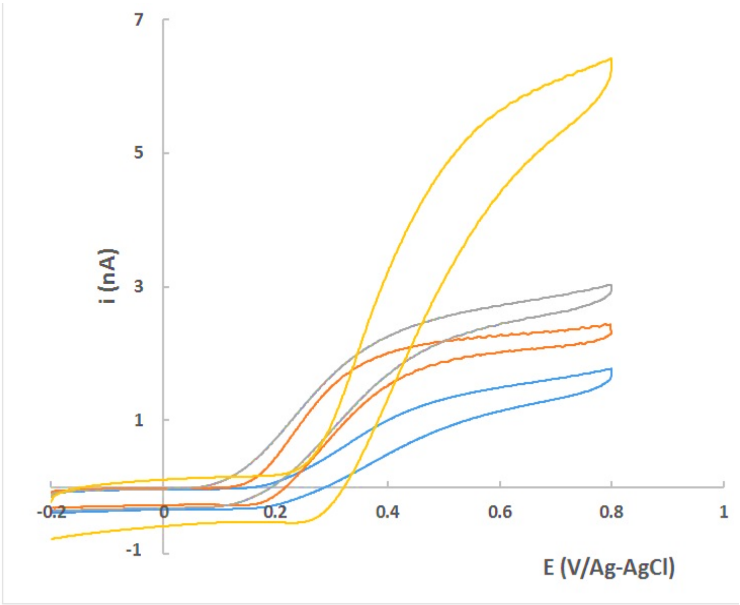

Figure S2. Representative cyclic voltammograms from a bare CFE (D:33 μm) (Blue line) and CNT/CFEs after electrodeposition time of 50 s (Red), 100 s (Gray) and 200 s (Orange) in 100 μM dopamine in PBS (pH 7.4) (Scan rate:100 mV/s, -0.2 to 0.8 V vs Ag/AgCl).

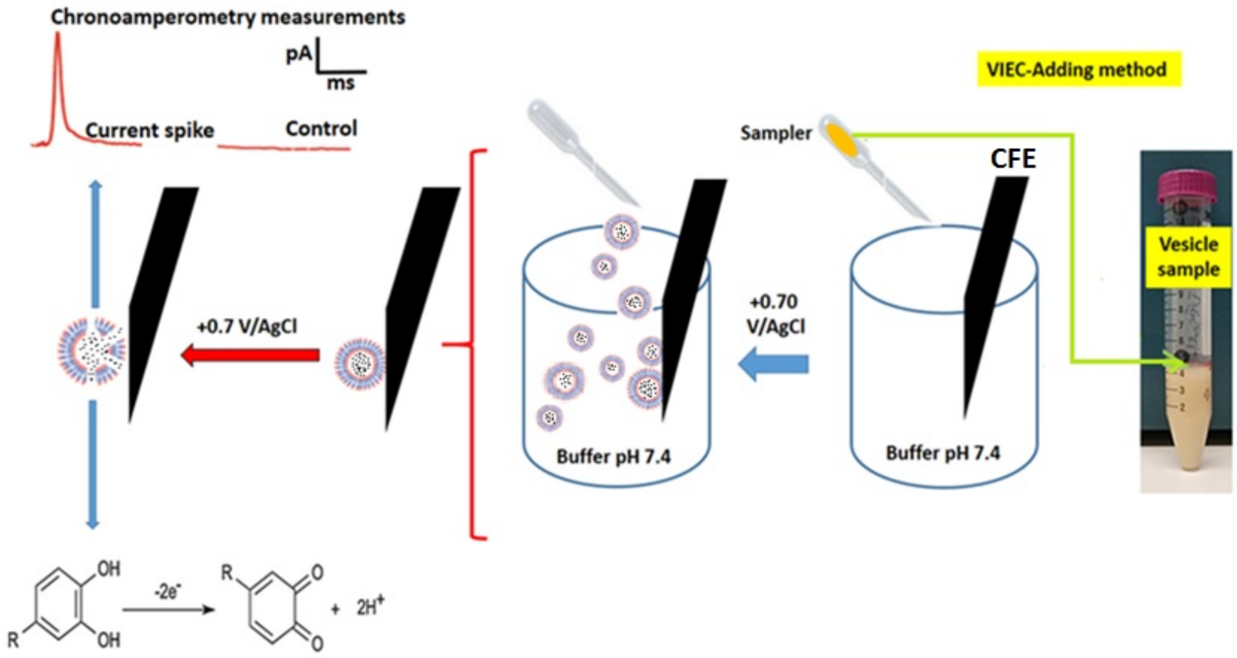

Figure S3. Schematic illustrating the experimental process of vesicle impact electrochemical cytometry (VIEC) using the adding technique.

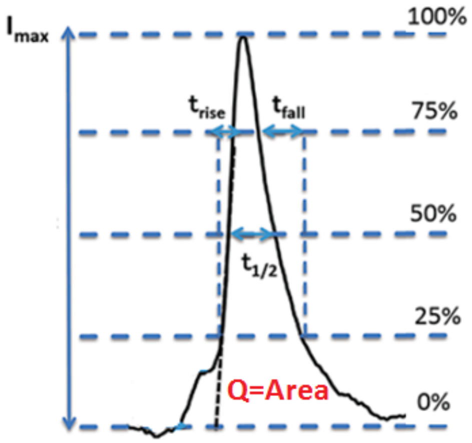

Figure S4. Scheme to show the different parameters for evaluation of representative spikes. Here, I_{max}: peak current, t_{rise}: rise time, $t_{1/2}$: half peak width, t_{fall}: fall time and Q: peak area: charge transferred (coulombs).

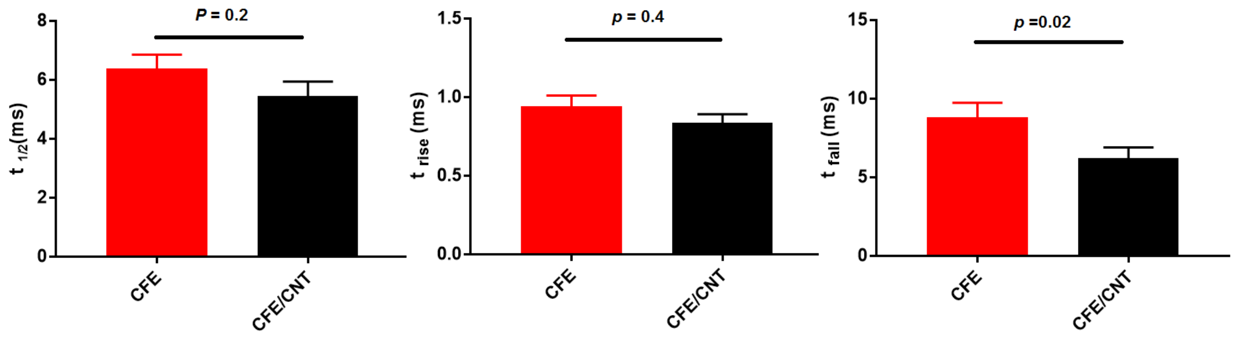

Figure S5. Experimental results for VIEC measurements by using CFE/CNTs and CFE. (A) $t_{1/2}$ (B) t_{rise}, and (C) t_{fall}. The data are presented as mean of medians. Error is the standard error of the mean (SEM). The pairs of data sets were compared using Wilcoxon−Mann−Whitney test. Error is the standard error of the mean (SEM).

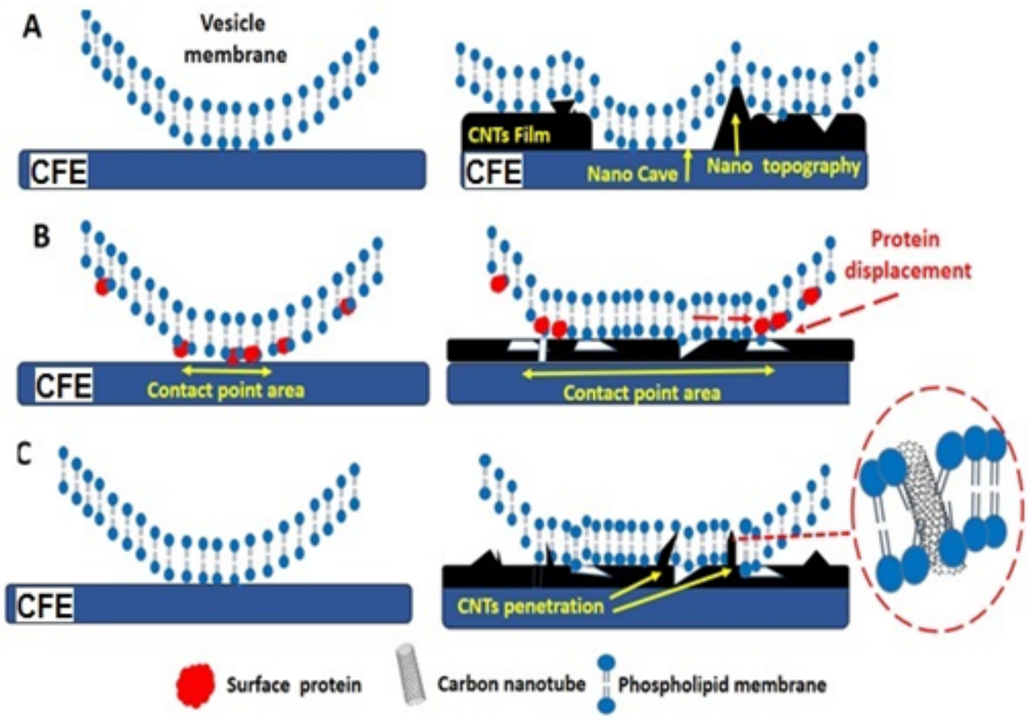

Figure S6. Possible models for the interaction between CNTs and an adsorbed vesicle. (A) Nano topography comparing a CFE to a CNTs/CFE surface showing the latter might induce nanoscale membrane curvature and bending. (B) Strong hydrophobic properties of CNTs might induce enhancement of the contact area between a vesicle and the electrode surface and surface proteins displacement could then occur after interaction between CNTs and the membrane. (C) Some CNTs might penetrate into the membrane and disturb the membrane structure.

References

1. Li, X. C.; Dunevall, J.; Ewing, A. G. *Faraday Discuss.* **2018,** *210*, 353-364.
2. Dunevall, J.; Fathali, H.; Najafinobar, N.; Lovric, J.; Wigstrom, J.; Cans, A. S.; Ewing, A. G. *J. Am. Chem. Soc.* **2015**, *137*, 4344-4346
3. Lovric, J.; Najaiinobar, N.; Dunevall, J.; Majdi, S.; Svir, I.; Oleinick, A.; Amatore, C.; Ewing, A. G., *Faraday Discuss.* **2016,** *193*, 65-79.
4. Hwang, J. Y.; Shin, U. S.; Jang, W. C.; Hyun, J. K.; Wall, I. B.; Kim, H. W. *Nanoscale,* **2013**, *5*, 487-497.
